# Influence of Humidity and Heating Rate on the Continuous ZIF Coating during Hydrothermal Growth

**DOI:** 10.3390/membranes13040414

**Published:** 2023-04-06

**Authors:** Eunji Choi, Choong-Hoo Lee, Dae Woo Kim

**Affiliations:** Department of Chemical and Biomolecular Engineering, Yonsei University, Yonsei-ro 50, Seodaemun-gu, Seoul 03722, Republic of Korea

**Keywords:** zeolitic imidazolate framework, membrane, scale-up, relative humidity, solution heating rate

## Abstract

Zeolitic imidazolate frameworks (ZIFs) have potential for various gas and ion separations due to their well-defined pore structure and relatively easy fabrication process compared to other metal–organic frameworks and zeolites. As a result, many reports have focused on preparing polycrystalline and continuous ZIF layers on porous supports with good separation performance in various target gases, such as hydrogen extraction and propane/propylene separation. To utilize the separation properties in industry, membrane is required to be prepared in large scale with high reproducibility. In this study, we investigated how humidity and chamber temperature influence the structure of a ZIF-8 layer prepared by the hydrothermal method. Many synthesis conditions can affect the morphology of polycrystalline ZIF membranes, and previous studies have mainly focused on reaction solutions, such as precursor molar ratio, concentration, temperature, and growth time. On the other hand, we found that the humidity of the chamber and the heating rate of the solution also lead to dramatic changes in the morphology of ZIF membranes. To analyze the trend between humidity and chamber temperature, we set up the chamber temperature (ranging from 50 °C to 70 °C) and relative humidity (ranging from 20% to 100%) using a thermo-hygrostat chamber. We found that as the chamber temperature increased, ZIF-8 preferentially grew into particles rather than forming a continuous polycrystalline layer. By measuring the temperature of the reacting solution based on chamber humidity, we discovered that the heating rate of the reacting solution varied with humidity, even at the same chamber temperature. At a higher humidity, the thermal energy transfer was accelerated as the water vapor delivered more energy to the reacting solution. Therefore, a continuous ZIF-8 layer could be formed more easily at low humidity ranges (ranging from 20% to 40%), while micron ZIF-8 particles were synthesized at a high heating rate. Similarly, under higher temperatures (above 50 °C), the thermal energy transfer was increased, leading to sporadic crystal growth. The observed results were obtained with a controlled molar ratio, in which zinc nitrate hexahydrate and 2-MIM were dissolved in DI water at a molar ratio of 1:45. While the results are limited to these specific growth conditions, our study suggests that controlling the heating rate of the reaction solution is critical for preparing a continuous and large-area ZIF-8 layer, particularly for the future scale-up of ZIF-8 membranes. Additionally, humidity is an important factor in forming the ZIF-8 layer, as the heating rate of the reaction solution can vary even at the same chamber temperature. Further research related to humidity will be necessary for the development of large-area ZIF-8 membranes.

## 1. Introduction

Metal–organic frameworks (MOFs) are a promising class of porous materials for separation applications, such as adsorbents and membranes, due to their well-defined crystalline pore structure [1,2,3]. Membrane separation offers several advantages over other separation methods, including energy efficiency, high selectivity, continuous processing, compact and modular design, mild operation conditions, and low maintenance requirements. These features bring advantages for various industries and applications, particularly for separating molecules with similar sizes, such as hydrocarbon separations. Membrane separation processes generally require less energy compared to other separation methods, such as distillation or evaporation, because they rely on concentration gradients or transmembrane pressures rather than on phase changes, which require significant energy input. In addition, when a membrane has a porous crystalline structure and ultrathin thickness, molecules can be separated precisely via molecular sieving with high permeance and selectivity.

One of the representative membrane material that can sieve gas molecules is zeolite due to its well-ordered pore structure and high stability in harsh operation conditions (high temperature and reactive gas), and significant progress has been made in recent years to improve the separation performance of zeolite membrane as well as to increase membrane size. Nonetheless, fabricating zeolite membranes poses challenges due to factors, such as precise crystal growth control, defect minimization, thin film fabrication, ceramic support preparation, and scalability and reproducibility. Defects, such as cracks, pinholes, or nonzeolitic pathways, can form during fabrication particularly during thermal activation, compromising membrane selectivity and performance. Therefore, careful synthesis and processing are required to minimize defects. Producing a thin, defect-free zeolite layer on a porous support is also challenging, as thin membranes are needed for high permeance and selectivity. This demands precise control of the seed layer on the porous support and growth conditions during secondary growth. Furthermore, good compatibility between the zeolite layer and the underlying support is crucial for mechanical stability and performance, which can be difficult due to differences in thermal expansion coefficients and chemical compatibility.

Compared to zeolites, MOFs can be fabricated into membranes using a relatively mild fabrication process [4,5]. MOF membranes are easier to fabricate than zeolite membranes due to factors, such as structural diversity, modularity, mild synthesis conditions, versatility in fabrication methods, and defect control. The wide range of structures and compositions in MOFs allows for tailoring membranes for specific applications. The modularity of MOFs enables precise control over pore size, shape, and functionality, simplifying the synthesis of membranes with the desired separation properties. MOF membranes can be synthesized under mild conditions, such as room temperature and atmospheric pressure, in contrast to the more demanding conditions for zeolite synthesis. In particular, MOFs do not require structure-guiding agents; therefore, an additional activation process is not required, and polymer support can be used as a support. However, low stability in harsh conditions, such as structure collapse and thermal degradation, must be addressed to be adopted in industry and many applications.

Among the subclasses of MOFs, the zeolitic imidazolate framework (ZIF) has received widespread attention because ZIF has a similar topology to zeolites with tetrahedral transition metal ions linked to imidazole-based ligands [6]. In particular, ZIF-8, which is constructed from Zn^2+^ and 2-methylimidazole, is the most extensively studied ZIF material because its aperture size is suitable for hydrocarbon separation and hydrogen extraction, and the aperture size can be easily tuned by ligand exchange or nanomaterial hybridization [7,8,9,10]. Therefore, significant efforts have been made to fabricate continuous ZIF-8 membranes, such as in situ synthesis (hydrothermal or solvothermal), contradiffusion synthesis, epitaxial synthesis, oxide conversion, electrochemical deposition, and fluid processing [11,12,13,14,15,16,17].

Among the aforementioned preparation methods, in situ synthesis (hydrothermal or solvothermal) is one of the common methods used for the synthesis of ZIF membranes due to its simplicity, scalability, and reproducibility [18,19,20,21]. In this method, a mixture of precursor solutions containing metal ions and organic ligands is placed in a bottle and heated at elevated temperatures in the presence of water or other solvents. The resulting conditions create an environment that promotes the formation and growth of ZIF crystals on a porous substrate, which serves as the support for the ZIF membrane [21]. When the nucleated crystals are merged into a polycrystalline layer, membrane fabrication is achieved. However, even if the fabrication process is simple, the quality of the grown ZIF is highly sensitive to growth conditions, and the presence of small defects results in poor gas separation performance.

Previous research has identified several factors that affect the microstructure and quality of synthesized ZIF-8 membranes, such as the nature of the zinc precursors (e.g., zinc nitrate, chloride, and acetate), solvent types (including polarity and precursor–solvent interactions), concentrations, and molar ratio of metal ion/organic ligands [22,23,24,25,26]. In addition to these factors, we investigated the effect of relative humidity and chamber temperature on the structure of the ZIF-8 layers formed during hydrothermal growth. We used a thermo-hygrostat chamber to control the temperature and relative humidity while conducting hydrothermal growth of ZIF-8 for one hour and recording the heating rate of the reacting solutions. We analyzed the morphology, crystallinity, and size distribution of the synthesized ZIF-8 particles under various reaction temperatures and relative humidity conditions.

## 2. Materials and Methods

### 2.1. Materials

Zinc nitrate hexahydrate (Zn(NO_3_)·6H_2_O, 98%, Sigma-Aldrich, St. Louis, MO, USA) and 2-methylimidazole (2-MIM, 99.0%, Sigma-Aldrich) were used as the metal and ligand sources. Glass substrate (Duran, 18 mm × 18 mm) was used for ZIF-8 growth. All the chemicals and materials were used as purchased without further purification.

### 2.2. Synthesis of ZIF-8 Layer with Different Humidity and Temperature

**Scheme 1:**
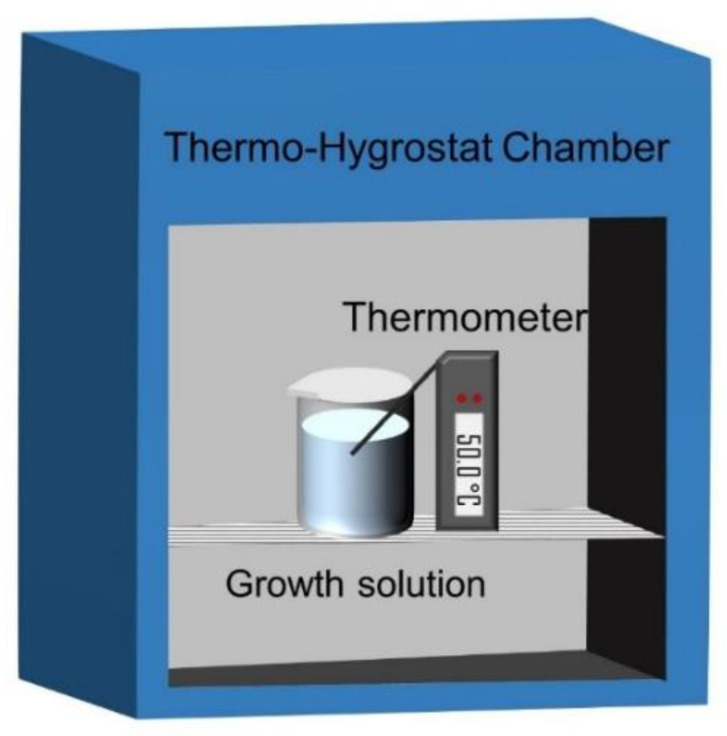
Schematic for the experiment set-up for ZIF-8 growth.

To synthesize a ZIF-8 layer, zinc nitrate hexahydrate (5.5 g) and 2-MIM (105.5 g) were dissolved separately in 1 L of DI water to obtain zinc ion and 2-MIM solutions, respectively. Then, 100 mL of each solution was mixed at room temperature in a molar ratio of 1:45, which has been widely reported as optimal for continuous ZIF membrane growth [7,8]. This paper used a fixed metal to ligand ratio for synthesizing ZIF-8 thin films due to the great impact of this ratio on the synthesis process. However, future studies will be conducted to investigate controlling the ratio and the effects of humidity. This concentration condition of the precursor solutions was chosen because of rapid crystal growth in the early stage. We note that the solvent can change the growth kinetics and morphology of ZIF particles; therefore, only water was used for this study. The mixed solution was placed in a thermo-hygrostat chamber as shown in Scheme 1, where temperature and relative humidity were precisely controlled. After stabilizing the temperature and relative humidity in the chamber by waiting several hours, the precursor solution was placed into the chamber. The temperature of the precursor solution was directly measured using a thermometer. The glass substrate was then immersed in the solution for 1 h to allow ZIF-8 growth. Afterward, the substrate was immersed in DI water to remove any unreacted precursors and overgrown ZIF-8 particles. Finally, the synthesized ZIF-8 layer was dried at 50 °C overnight.

### 2.3. Characterization

The morphology of ZIF-8 crystal was observed using scanning electron microscopy (SEM, 7610F-PLUS, JEOL, Akishima, Japan) and X-ray diffraction (XRD) patterns by a film XRD system (Ultima IV, Rigaku, Tokyo, Japan, 3 kW sealed Cu Kα X-ray source, λ = 1.5406 Å). Fourier Transform Infrared Spectroscopy (FT-IR, ALPHA II, Bruker, Billerica, MA, USA) was used to determine the chemical bonding of the ZIF-8 films and particles.

### 2.4. Measuring the Size Distribution of ZIF-8 Crystal Grain Size

**Figure 1:**
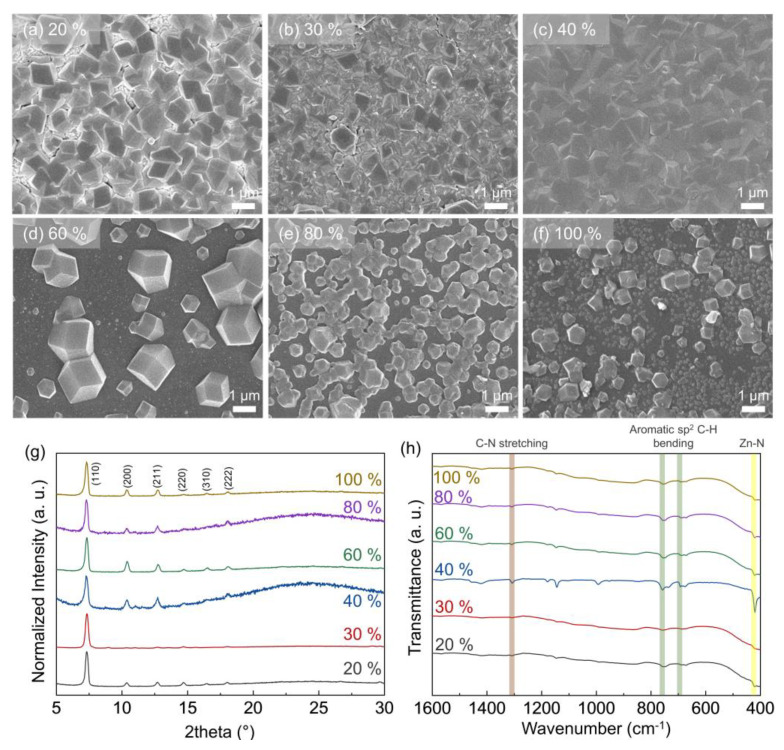
(**a**–**f**) SEM images ((**a**): 20%, (**b**): 30%, (**c**): 40%, (**d**): 60%, (**e**): 80%, and (**f**): 100%). (**g**) XRD patterns and (**h**) FT-IR spectra of ZIF-8 crystals depending on relative humidity (20, 30, 40, 60, 80, and 100%). The temperature of the chamber was 50 °C.

**Figure 2:**
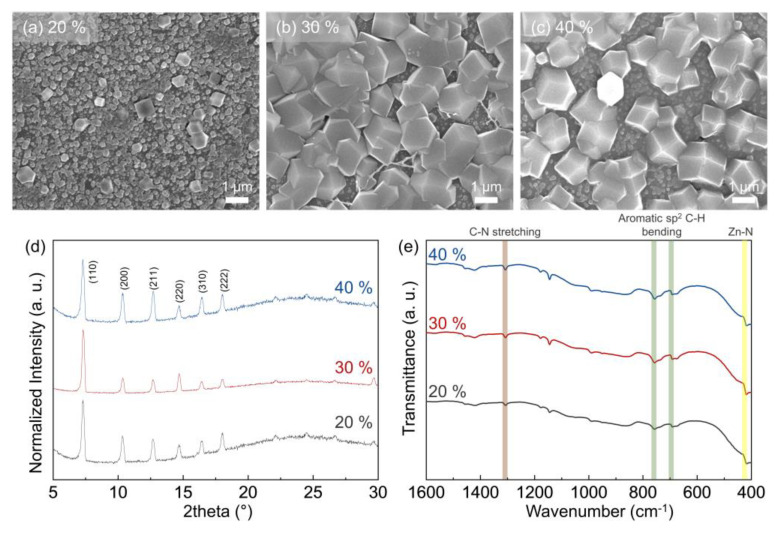
(**a**–**c**) SEM images ((**a**): 20%, (**b**): 30%, and (**c**): 40%), (**d**) XRD patterns, and (**e**) FT-IR spectra for the ZIF-8 grown on the substrates depending on relative humidity (20, 30, and 40%) at chamber temperature of 60 °C.

**Figure 3:**
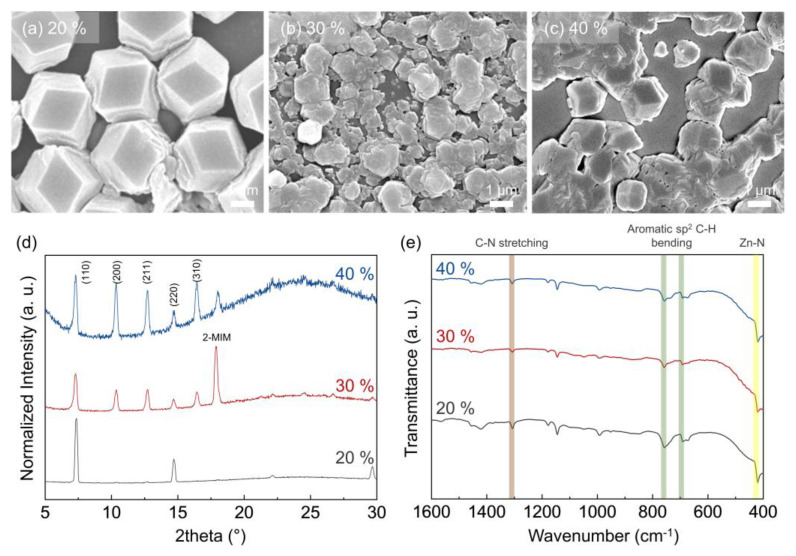
(**a**−**c**) SEM images ((**a**): 20%, (**b**): 30%, and (**c**): 40%), (**d**) XRD patterns, and (**e**) FT-IR spectra for the ZIF-8 grown on the substrates depending on relative humidity (20, 30, and 40%) at chamber temperature of 70 °C.

Based on the SEM images, such as those shown in Figure 1, Figure 2 and Figure 3, the grain size of synthesized ZIF-8 crystals was measured by using the ImageJ Program (Version: 64-bit Java 8). A total of 1000 counts per image were made to reveal the size distribution of the ZIF-8 grains and crystals. For images with less than 1000 individual crystal grains, all the crystals were counted. The size of the grains was defined as the largest diameter of the particles and grains, even if the grain’s shape was not circular.

## 3. Results and Discussions

### 3.1. Morphology of ZIF-8 Crystals Depending on Chamber Temperature and Relative Humidity

The hydrothermal method is widely used to synthesize ZIF-8 membranes due to its ability to provide controlled growth conditions, resulting in high-quality crystals. It is a scalable and reproducible method that is relatively simple and cost-effective compared to other methods. ZIF-8 membranes with excellent separation performance have been reported using the hydrothermal method [7,8]. In this study, we focused on the growth of ZIF-8 and the effect of growth conditions, specifically the relative humidity and temperature of the chamber, while keeping other growth conditions identical, such as growth time (1 h). Water was used as a solvent to avoid the solvent effect on the nucleation and growth of ZIF-8. We used a cover glass substrate for ZIF-8 growth, which was chosen for its simplicity and convenience in the ZIF-8 fabrication, as well as its similar chemical structure to conventional ceramic substrates, such as alumina and silica, with oxygen groups [27]. Firstly, ZIF-8 growth on a glass substrate was observed at 50 °C by varying the relative humidity from 20% to 100%, and the morphology and crystal structure were investigated (Figure 1). There were two standards to confirm that the synthesized ZIF-8 is either polycrystalline ZIF-8 film or ZIF-8 particles. First, the interval between synthesized crystals should be less than several hundred nanometers at least. Second, the distinct facet of ZIF-8 crystal should be observed. Our results show that ZIF-8 films were continuous and highly crystalline at relative humidities between 20% and 40% (Figure 1a–c). However, at higher relative humidities, ZIF-8 crystals formed as island particles rather than a continuous film (Figure 1d–f). Notably, we observed large and highly crystalline ZIF-8 particles at 60% relative humidity, while small ZIF-8 particles were present above 80% relative humidity.

The crystal structure of the ZIF-8 layer or particles was investigated using XRD, showing the major XRD six peaks of ZIF-8 within 20 degrees without clear differences (Figure 1g). In particular, under 60 to 100% relative humidity, while there was a noticeable difference (polycrystalline or island growth) in the SEM images, no significant changes were detected in the XRD patterns. This means that the ZIF-8 crystal was well constructed, but the morphology of the grown ZIF-8 particles was different depending on the reaction conditions. The FT-IR investigation of ZIF-8 films or particles was also performed. ZIF-8 peaks, such as the stretching vibration of the Zn–N bond at 426 cm^−1^, the aromatic sp^2^ bending of 2-MIM at 678 and 737 cm^−1^, and the stretching of the C–N bond at 1310 cm^−1^, were observed [28]. These results indicate that the morphology of grown ZIF-8 is highly influenced by the relative humidity of the chamber, while other conditions were identical. However, their crystalline structures at varying relative humidity were identical at 50 °C of the chamber temperature.

In order to investigate the effect of the temperature and relative humidity during ZIF-8 growth, the chamber temperature was increased from 50 °C to 60 °C with relative humidities ranging from 20% to 40% (Figure 2). Continuous ZIF-8 layers were not formed at 60 °C, with the particle size varying depending on the relative humidity of the chamber. At 60 °C and 20% humidity, the ZIF-8 particle size was around 400–500 nm (Figure 2a). Increasing the relative humidity led to a significant increase in particle size, up to 1 μm (Figure 2b,c). Unlike ZIF-8 particles with a high relative humidity at 50 °C, these ZIF-8 particles at 60 °C exhibited high crystallinity and chemical bonding with distinct facets of ZIF-8, possibly due to their large size. Furthermore, the relative ratio of the XRD peaks for the synthesized ZIF-8 layer was found to be different from that of the ZIF-8 layer synthesized at 50 °C, with relatively high intensities observed for the (200) and (211) peaks of ZIF-8 layer, as shown in Figure 2d. This XRD result indicates the partial change of orientation of the ZIF-8 grains as the chamber temperature changes. Additionally, no discernible differences in the chemical bonding of the synthesized ZIF-8 were observed, as depicted in Figure 2e. It is known that the deprotonation of 2-MIM can be facilitated at higher temperatures [29], leading to more nucleation of ZIF-8 seeds. However, the growth of relatively larger ZIF-8 particles at higher temperatures can be attributed to more facilitated particle growth than nucleation.

The ZIF-8 particles were synthesized at 70 °C under relative humidity conditions ranging from 20% to 40% to investigate their growth at higher temperatures (Figure 3). Uniform and microsized particles were observed at 20% relative humidity, as shown in Figure 3a. In contrast, ZIF-8 particles synthesized at 30% and 40% relative humidity appeared as clusters with low crystallinity, and the grain boundary of the ZIF-8 crystal was not observed clearly in Figure 3b,c. Interestingly, the XRD peaks of the ZIF-8 particles synthesized at 20% relative humidity were very sharp with a preferential (110)-orientation. This observation was confirmed by the relative intensity of the (110) peaks being higher than other facet peaks of ZIF-8, which was also correlated with the SEM image in Figure 3a displaying the oriented ZIF-8 facets. While the reason is not clear at this point, the preferential orientation of ZIF-8 is interesting. Because this study is not focused on crystalline orientation, a further study will be reported soon. The diffraction of 2-MIM for the ZIF-8 particles synthesized at 30% and 40% was strongly observed near 18 degrees, indicating the growth of ZIF-8 particles by combining with 2-MIM, as shown in Figure 3d. The SEM images in Figure 3b,c did not show clear facets and grain boundaries of the ZIF-8 crystals, and the weak (110) XRD peak of the synthesized ZIF-8 layer was observed in Figure 3d, compared to the ZIF-8 synthesized at 50 °C and 60 °C. These SEM and XRD data proved that the synthesized ZIF-8 was formed as clusters with remaining 2-MIM rather than distinct ZIF-8 crystals. In other words, the synthesized ZIF-8 at 30 and 40% grew by agglomeration rather than by well-defined crystal growth mechanisms. However, several FT-IR spectra in Figure 3e for ZIF-8 were observed at this temperature and relative humidity, indicating no significant change in the chemical bonding of ZIF-8 and 2-MIM.

### 3.2. Particle and Grain Size Distribution

**Figure 4:**
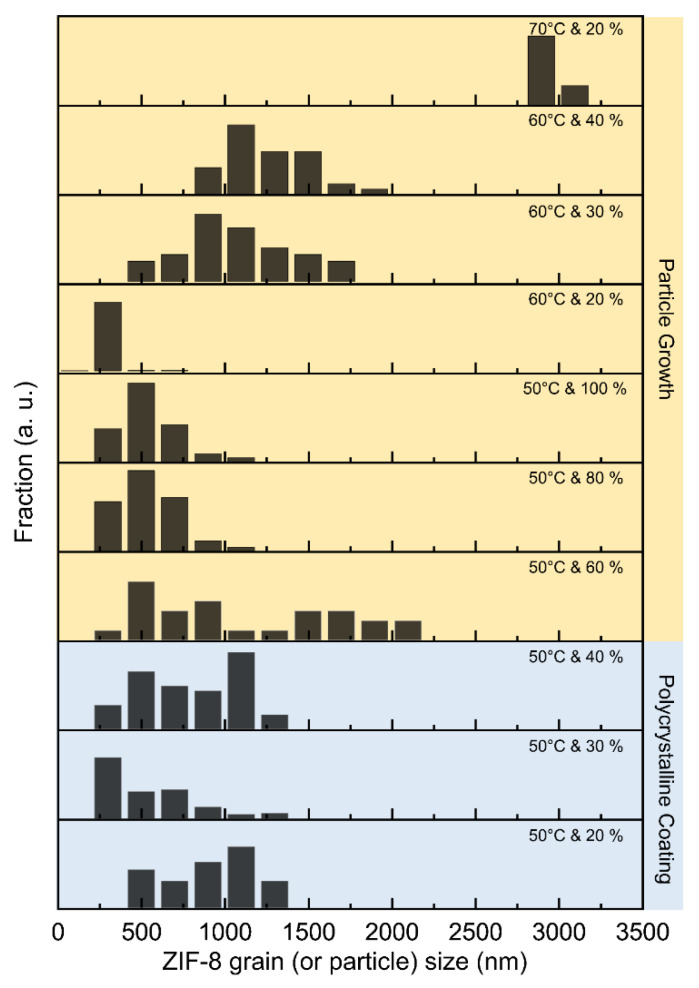
ZIF-8 grain (or particle) size distribution depending on the temperature and relative humidity. Because crystallinity of ZIF-8 was not high at the relative humidity (30 and 40%) and 70 °C, the grain size was not measured.

The effects of different temperature and relative humidity conditions on the size of the ZIF-8 grains or particles are summarized in Figure 4. The grain size distribution was obtained by measuring the lateral size of each ZIF-8 grain observed in the SEM images. For a reliable analysis, approximately 1000 grains were counted for each condition. For polycrystalline ZIF-8 coating (sky blue area), most of the grain sizes ranged from 500 to 1200 nm, and no significant changes in grain size were observed. However, at a constant temperature of 50 °C, an increase in relative humidity (60%) led to an increase in the grain size of the ZIF-8 particles, reaching several micrometers from nanometers, followed by a dramatic decrease in grain size ranging from 300 to 700 nm, particularly at 80% and 100% relative humidity. Moreover, the particle size of the ZIF-8 increased (yellow area) to several micrometers at 60 °C and 70 °C, regardless of the relative humidity. Notably, the ZIF-8 particles at 70 °C consistently had a large particle size of about 3 μm with a highly oriented crystal structure. Because the ZIF-8 layer used for the membrane fabrication is very thin (submicrometer thickness), the formation of micrometer-scale particles is not suitable for producing a continuous polycrystalline ZIF-8 layer. Therefore, the importance of optimizing conditions, such as temperature and relative humidity, might be increased.

**Figure 5:**
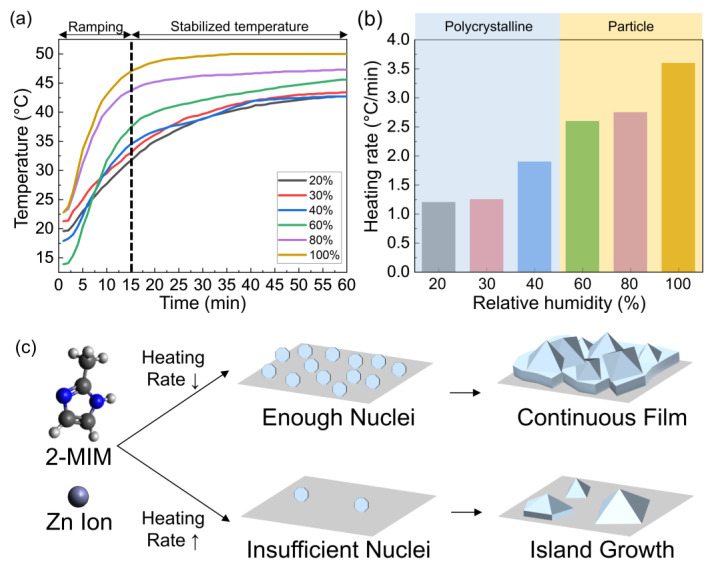
(**a**) Temperature profile and (**b**) Heating rate graphs of the reacting solutions during ZIF-8 growth at 50 °C for different relative humidities. (**c**) Schematic diagram of the growth mechanism of ZIF-8 crystals depending on the heating rate of the reaction solution.

To investigate the relationship between relative humidity and the growth mechanism of ZIF-8, the temperature profiles of the growth solution were measured at a chamber temperature of 50 °C for several humidity ranges. The temperature in the reacting solution was measured using a portable thermometer (Scheme 1). Figure 5a shows that the temperature of all solutions gradually increased in the first 15 min and then stabilized, indicating two different heating rate trends. The heating rate depending on the relative humidity was calculated in Figure 5b, which was determined from the slope of Figure 5a. The heating rate was similar at a low humidity (20–40%), ranging from 1 to 2 °C/min, but it dramatically increased at a high humidity (60–100%), ranging from 2.5 to 3.5 °C/min.

The presence of water vapor in the air can enhance thermal energy transfer due to the increased heat capacity of water molecules compared to dry air. This means that water molecules require more energy to raise their temperature, which can then be transferred to other objects or surfaces through convection or conduction. Therefore, even if the chamber temperature is the same, the real temperature of the reacting solution can significantly differ depending on the amount of relative humidity. Due to that, competitive reactions between nucleation and the crystal growth of ZIF-8 occurred in the reacting solution, leading to different growth mechanisms and kinetics. Based on previous studies on ZIF-8 particle growth in solution, sometimes increasing the growth temperature can lead to an increase in the size of the ZIF-8 particles because higher temperatures can increase the growth rate of the ZIF-8 crystal [29]. On the other hand, high temperatures can also facilitate the deprotonation of 2-MIM, which is favorable to form nuclei, leading to a small particle size [30,31]. In addition, excessively high temperatures (above 70 °C) may not be suitable for ZIF-8 crystal formation. High temperatures can lead to rapid nucleation and growth rates, which in turn can cause the formation of crystals with reduced crystallinity. It is likely that the accelerated process may not allow enough time for the proper arrangement of the organic linkers and metal ions. In addition, temperatures close to the boiling point of the solvent may induce solvent evaporation, resulting in nonuniform crystal growth and the formation of amorphous materials. Therefore, a continuous ZIF-8 coating process is highly complicated with various factors.

To summarize, at a fixed temperature of 50 °C, the formation of enough ZIF-8 nuclei resulted in the formation of a polycrystalline film with a uniform grain size of 500–1200 nm at low heating rates of the reacting solution. However, at high heating rates (Figure 5c), insufficient ZIF-8 nuclei were formed, leading to island growth of the ZIF-8 crystals with varying particle sizes. Furthermore, island growth with low crystallinity was also observed at higher temperatures of 60 °C and 70 °C. The precise relationship between growth temperature and particle size can vary depending on other factors, such as precursor concentration, solvent, reaction time, and even substrate [32,33]. Therefore, the critical temperature and relative humidity range suitable for membrane fabrication may differ based on the experimental conditions and environmental factors. In particular, the molar ratio of organic linker and metal ion is crucial for successful MOF synthesis because it affects the crystallinity, phase purity, pore size, functionality, stability, yield, and synthesis efficiency of the resulting MOFs. Therefore, detailed temperature conditions must be carefully considered depending on the growth conditions. Nonetheless, our observations highlight the significance of considering chamber temperature and relative humidity, which have not been given much importance previously.

## 4. Conclusions

Large-scale membrane fabrication is important due to its impact on economies of scale, commercial viability, and technology advancement. Producing membranes on a large scale reduces the membrane fabrication cost, accelerating the technology adoption in various industries, such as water treatment, gas separation, pharmaceuticals, and energy production. This scalability will make opportunities to drive further research and development, leading to new materials, improved fabrication methods, and enhanced performance, thereby overcoming existing limitations and expanding membrane applications. Even if there are many reports on the fabrication of high-performance membranes with MOFs, fabrication was commonly achieved on a lab scale or in a small area. Therefore, there are growing demands to find a better way to make the membrane in large scale by reproducible methods. We demonstrated that the morphology of ZIF-8 can be dramatically influenced by the relative humidity and temperature of the chamber that is used for hydrothermal synthesis. Generally, the temperature of the chamber is considered as the growth condition, but the temperature change of the growth solution has a significant impact on the growth of ZIFs rather than on the temperature of the chamber. To explore the impact of different parameters on ZIF-8 growth, we employed a well-controlled thermo-hygrostat chamber during the ZIF-8 growth reaction. During the in situ growth of ZIF-8 in this chamber, we observed the ZIF-8 nucleation in the early stages, but the number of ZIF-8 nuclei varied depending on the temperature and relative humidity. As a result, different types of ZIF-8 films or particles formed. In particular, the heating rate of the reacting solutions varied accordingly; therefore, it is highly important to manipulate the heating rate or relative humidity for specific applications. Within the scope of the studied conditions, the heating rate should be maintained below 2.5 °C/min for continuous film growth. Furthermore, when using higher temperatures (above 50 °C), thermal energy transfer is excessively high to yield continuous films. Our study provides the first comprehensive report on the relationship between relative humidity and ZIF-8 growth tendencies. In particular, this phenomenon is crucial for the future manufacturing of large-area membranes. For large-scale synthesis, it is necessary to uniformly control the solvent temperature to ensure a reproducible membrane manufacturing process. Although the observed growth conditions are limited to the hydrothermal process, it implies that humidity and temperature control may also be important in other fabrication methods.

## Data Availability

Not applicable.

## References

[1] Wang H., Dong X., Colombo V., Wang Q., Liu Y., Liu W., Wang X.L., Huang X.Y., Proserpio D.M., Sironi A. (2018). Tailor-Made Microporous Metal–Organic Frameworks for the Full Separation of Propane from Propylene Through Selective Size Exclusion. Adv. Mater..

[2] Cui W.G., Hu T.L., Bu X.H. (2019). Metal–Organic Framework Materials for the Separation and Purification of Light Hydrocarbons. Adv. Mater..

[3] Yuan S., Feng L., Wang K., Pang J., Bosch M., Lollar C., Sun Y., Qin J., Yang X., Zhang P. (2018). Stable Metal–Organic Frameworks: Design, Synthesis, and Applications. Adv. Mater..

[4] Wang H., Liu Y., Li J. (2020). Designer Metal–Organic Frameworks for Size-Exclusion-Based Hydrocarbon Separations: Progress and Challenges. Adv. Mater..

[5] Li J., Bhatt P.M., Li J., Eddaoudi M., Liu Y. (2020). Recent Progress on Microfine Design of Metal–Organic Frameworks: Structure Regulation and Gas Sorption and Separation. Adv. Mater..

[6] Krokidas P., Moncho S., Brothers E.N., Castier M., Economou I.G. (2018). Tailoring the gas separation efficiency of metal organic framework ZIF-8 through metal substitution: A computational study. Phys. Chem. Chem. Phys..

[7] Choi E., Hong S.J., Kim Y.J., Choi S.E., Choi Y., Kim J.H., Kang J., Kwon O., Eum K., Han B. (2021). Pore Tuning of Metal-Organic Framework Membrane Anchored on Graphene-Oxide Nanoribbon. Adv. Funct. Mater..

[8] Choi E., Choi J.I., Kim Y.J., Kim Y.J., Eum K., Choi Y., Kwon O., Kim M., Choi W., Ji H. (2022). Graphene Nanoribbon Hybridization of Zeolitic Imidazolate Framework Membranes for Intrinsic Molecular Separation. Angew. Chem. Int. Ed..

[9] Fan W., Ying Y., Peh S.B., Yuan H., Yang Z., Yuan Y.D., Shi D., Yu X., Kang C., Zhao D. (2021). Multivariate Polycrystalline Metal–Organic Framework Membranes for CO_2_/CH_4_ Separation. J. Am. Chem. Soc..

[10] Lee M.J., Kwon H.T., Jeong H.K. (2017). High-Flux Zeolitic Imidazolate Framework Membranes for Propylene/Propane Separation by Postsynthetic Linker Exchange. Angew. Chem. Int. Ed..

[11] Zhou Y., Zhang X.-F., Yao J., Wang H. (2022). Contra-diffusion synthesis of metal-organic framework separation membranes: A review. Sep. Pur. Technol..

[12] Bux H., Liang F., Li Y., Cravillon J., Wiebcke M., Caro J. (2009). Zeolitic imidazolate framework membrane with molecular sieving properties by microwave-assisted solvothermal synthesis. J. Am. Chem. Soc..

[13] Yeo Z.Y., Zhu P.W., Mohamed A.R., Chai S.-P. (2014). A well inter-grown ZIF-8 membrane synthesized via two-step hydrothermal synthesis on coarse α-Al_2_O_3_ support. Mater. Lett..

[14] Eum K., Rownaghi A., Choi D., Bhave R.R., Jones C.W., Nair S. (2016). Fluidic Processing of High-Performance ZIF-8 Membranes on Polymeric Hollow Fibers: Mechanistic Insights and Microstructure Control. Adv. Funct. Mater..

[15] Zhao Y., Wei Y., Lyu L., Hou Q., Caro J., Wang H. (2020). Flexible Polypropylene-Supported ZIF-8 Membranes for Highly Efficient Propene/Propane Separation. J. Am. Chem. Soc..

[16] Neelakanda P., Barankova E., Peinemann K.-V. (2016). Polymer supported ZIF-8 membranes by conversion of sputtered zinc oxide layers. Microporous Mesoporous Mater..

[17] Valadez Sánchez E.P., Gliemann H., Haas-Santo K., Wöll C., Dittmeyer R. (2016). ZIF-8 SURMOF Membranes Synthesized by Au-Assisted Liquid Phase Epitaxy for Application in Gas Separation. Chem. Ing. Tech..

[18] Kwon H.T., Jeong H.-K. (2013). In Situ Synthesis of Thin Zeolitic–Imidazolate Framework ZIF-8 Membranes Exhibiting Exceptionally High Propylene/Propane Separation. J. Am. Chem. Soc..

[19] Isaeva V.I., Barkova M.I., Kustov L.M., Syrtsova D.A., Efimova E.A., Teplyakov V.V. (2015). In situ synthesis of novel ZIF-8 membranes on polymeric and inorganic supports. J. Mater. Chem. A.

[20] Liu Y., Wang N., Pan J.H., Steinbach F., Caro J. (2014). In situ synthesis of MOF membranes on ZnAl-CO_3_ LDH buffer layer-modified substrates. J. Am. Chem. Soc..

[21] Shah M., Kwon H.T., Tran V., Sachdeva S., Jeong H.-K. (2013). One step in situ synthesis of supported zeolitic imidazolate framework ZIF-8 membranes: Role of sodium formate. Microporous Mesoporous Mater..

[22] He M., Yao J., Li L., Zhong Z., Chen F., Wang H. (2013). Aqueous solution synthesis of ZIF-8 films on a porous nylon substrate by contra-diffusion method. Microporous Mesoporous Mater..

[23] Ramu G., Lee M., Jeong H.-K. (2018). Effects of zinc salts on the microstructure and performance of zeolitic-imidazolate framework ZIF-8 membranes for propylene/propane separation. Microporous Mesoporous Mater..

[24] Watanabe S., Ohsaki S., Hanafusa T., Takada K., Tanaka H., Mae K., Miyahara M.T. (2017). Synthesis of zeolitic imidazolate framework-8 particles of controlled sizes, shapes, and gate adsorption characteristics using a central collision-type microreactor. Chem. Eng. J..

[25] Yao J., Li L., Benjamin Wong W.H., Tan C., Dong D., Wang H. (2013). Formation of ZIF-8 membranes and crystals in a diluted aqueous solution. Mater. Chem. Phys..

[26] Bustamante E.L., Fernández J.L., Zamaro J.M. (2014). Influence of the solvent in the synthesis of zeolitic imidazolate framework-8 (ZIF-8) nanocrystals at room temperature. J. Colloid Interface Sci..

[27] Nagai N., Hashimoto H. (2001). FT-IR-ATR study of depth profile of SiO_2_ ultra-thin films. Appl. Surf. Sci..

[28] Tanaka S., Tanaka Y. (2019). A Simple Step toward Enhancing Hydrothermal Stability of ZIF-8. ACS Omega.

[29] Saliba D., Ammar M., Rammal M., Al-Ghoul M., Hmadeh M. (2018). Crystal Growth of ZIF-8, ZIF-67, and Their Mixed-Metal Derivatives. J. Am. Chem. Soc..

[30] Wu R., Fan T., Chen J., Li Y. (2019). Synthetic Factors Affecting the Scalable Production of Zeolitic Imidazolate Frameworks. ACS Sustain. Chem. Eng..

[31] Xue Y., Zhao Q., Luan C. (2001). The Thermodynamic Relations between the Melting Point and the Size of Crystals. J. Colloid Interface Sci..

[32] Choi E., Lee J., Kim Y.-J., Kim H., Kim M., Hong J., Kang Y.C., Koo C.M., Kim D.W., Kim S.J. (2022). Enhanced Stability of Ti_3_C_2_T_x_ MXene Enabled by Continuous ZIF-8 Coating. Carbon.

[33] Kwon O., Kim M., Choi E., Bae J.H., Yoo S., Won J.C., Kim Y.H., Shin J.H., Lee J.S., Kim D.W. (2022). High-aspect Ratio Zeolite Framework (ZIF) Nanoplates for Hydrocarbon Separation Membranes. Sci. Adv..

